# Effects of emodin on ABC transporter gene expression in grass carp (*Ctenopharyngodon idellus*) exposed to diazinon

**DOI:** 10.1371/journal.pone.0219866

**Published:** 2019-07-31

**Authors:** Guihong Fu, Xuanyun Huang, Bo Qin, Yanqing Wu, Yuan Wang, Shu Zhao, Junfang Zhou, Wenhong Fang

**Affiliations:** Key Laboratory of East China Sea Fishery Resources Exploitation, Ministry of Agriculture and Rural Affairs; East China Sea Fisheries Research Institute, Chinese Academy of Fishery Sciences, Shanghai, China; Stazione Zoologica Anton Dohrn, ITALY

## Abstract

This study aimed to investigate the function of ATP-binding cassette (ABC) transporter genes in grass carp treated with emodin combined with diazinon (DZN) exposure. The transcription levels of five ABC transporter genes in different tissues of grass carp and at different time points were measured by real-time quantitative PCR (qRT-PCR). The analysis of different tissues showed higher *ABCB1* expression in the skin (26-fold) and gill (2-fold) than in the liver. In addition, *ABCB11* expression was higher in the skin (109-fold) and gill (57-fold) than in the liver, *ABCC1* was more highly expressed in the gill (50-fold) than in the liver, and *ABCG*2 was expressed at higher levels in the skin (659-fold, *p* < 0.01), gill (628-fold, *p* < 0.01) and liver (659-fold, *p* < 0.01) than in brain tissue. The analysis of different time points revealed that the *ABCB*1, *ABCB*11, *ABCC*1, *ABCC*2 and *ABCG*2 genes were highly expressed at 24 h in the liver in the experimental group. However, analysis of the intestinal tissue of the experimental group showed that the expression of *ABCB1* and *ABCB11* peaked at 6 h, the expression of *ABCC1* and *ABCC2* peaked at 5 d, and the expression of *ABCG2* peaked at 3 d. Furthermore, the emodin concentrations in the liver and intestine reached their peak levels (50.18 and 117.24 μg·ml^−1^, respectively) after 48 and 1 h of treatment with emodin combined with DZN, respectively. The peak DZN concentrations in the liver (1.42 ng·ml^−1^) and intestine (0.2 ng·ml^−1^) were detected 3 and 6 h after emodin treatment combined with DZN, respectively. In conclusion, this study shows that the transcript levels of ABC transporters respond to the presence of emodin, which indicates their potential involvement in and contribution with the metabolic process in grass carp.

## Introduction

The ATP-binding cassette (ABC) proteins are considered the first line of defense against environmental pollutants in organisms living in aquatic environments, which are the final destinations of most pollutants [1]. ABC transporter proteins are involved in substrate transport processes across biological membranes and are highly conserved transmembrane active transport proteins [2]. Furthermore, ABC transporters can transport environmental pollutants to aquatic animals. The family of animal ABC transporters, however, is far more extensive but shows high functional diversity. Examples of these ABC transporters are P-glycoprotein (P-gp*; ABCB*1), multidrug resistance-associated proteins 1–5 (MRP, *ABCC*1-5), and multixenobiotic resistance proteins (MXR, BCRP/*ABCG*2). In previous studies, various animal models have been used to indicate that ABCB, ABCC and ABCG are the most relevant proteins associated with resistance to toxic substances in aquatic organisms [3–5]. For example, *ABCB1* (P-gp), an ATP-dependent efflux pump, was first discovered in teleosts through a molecular genetic study of winter flounder (*Pleuronectes americanus*) [6]. *ABCC*1-5 are highly conserved ABC proteins in aquatic animals that might play a central role in adaptation in response to environmental stress by acting as xenobiotic transporters [7]. *ABCG*2 belongs to subfamily G, which plays a crucial role in this process in aquatic organisms [8]. ABC proteins consist of half and full transporters that possess one nucleotide-binding domain (NBD) and one transmembrane-binding domain (TMD) or two NDBs and two TMDs, respectively. The main difference between *ABCG2* and *ABCB*1/*ABCC*1-5 is that *ABCG*2 is considered a half transporter that possesses one NBD and one TMD, whereas ABCB1 and ABCC1-5 are full transporters with two NDBs and two TMDs. As a half transporter, *ABCG*2 needs to undergo dimerization before it can exhibit the ability to transport substrates, and thus, the substrate spectrum of *ABCG*2 is narrower than those of other ABC proteins. In addition, it is known that *ABCG*2 is involved in the excretion of physiological metabolites but not conjugated toxins [9]. The greater pressures experienced by teleost fishes in their aquatic environment, such as xenobiotic and toxicant stresses, complicates the characterization of teleost ABC transporters. Therefore, extensive studies are needed to investigate fish ABC transporters and to determine how their functions are influenced by drugs used in aquaculture.

Emodin (1,3,8-trihydroxy-6-methylanthraquinone), a natural anthraquinone derivative isolated from *Rheum palmatum* L., has been reported to potentiate the anti-proliferation of various chemotherapeutic agents [10]. In addition, emodin serves as an inhibitor of P-gp (ABCB1) because it inhibits P-gp efflux function and protein expression in Caco-2 cells [11]. It has also been hypothesized that emodin inhibits ABCB1 function by competitively binding to transport sites [12].

Organophosphorus pesticides (OPs), which are insecticides that are used worldwide and released into the environment, have the potential to exert adverse effects on wildlife populations [13]. Diazinon (DZN), an important OP that exerts potential broad-spectrum effects against different insect and parasite populations, is released into environmental and aquaculture waters [14, 15]. It is also known that DZN is extremely toxic to aquatic animals (Environmental Protection Agency (EPA) 2003). For example, DZN has been found in aquatic systems in Europe: low concentrations of DZN have been measured in the North Sea (up to 0.046 ng·L^−1^), and the highest concentration was detected in the Ebro River in Spain (up to 785 ng·L^−1^) [16]. The toxic effects of DZN on embryos and larvae of common carp were previously investigated, and the results showed that low concentrations of DZN (0.25 mg·kg^-1^) in aquatic environments exert a significant effect on the reproduction and development of common carp [17]. The effects of DZN on the behavior of zebrafish have been investigated [18]. However, DZN degrades rapidly under low temperature conditions and in the absence of suitable microbiological degraders, which remain biologically active in soils for at least six months. DZN affects a wide range of nontarget organisms in aquatic environments, such as invertebrates, mammals and fishes, particularly those inhabiting aquatic environments [19, 20]. To achieve this phenomenon, DZN can persist in water for 6 months and can thus exert dangerous cumulative effects on the tissues of aquatic animals [21]. Therefore, it is important to investigate the effects of DZN on aquatic animals, and we thus aimed to assess the effects of DZN exposure on grass carp in this study.

Grass carp (*Ctenopharyngodon idellus*), one of the most common freshwater fish species worldwide, exhibits the widest distribution of all farmed aquaculture species and shows great adaptability to virtually every environmental condition [22]. Grass carp is a good model for the assessment of aquatic ecosystems, and various toxicological studies, such as a study investigating the tissue accumulation and toxicity of isothiazolinone [23], have been performed using grass carp as the model organism.

The major objective of this study was to investigate the transcription levels of ABC transporters in grass carp after emodin treatment combined with DZN exposure. Furthermore, the mRNA expression of ABC transporters and the concentrations of emodin and DZN in several tissues of grass carp were measured at different time points after emodin treatment combined with DZN exposure.

## Materials and methods

### Chemicals and reagents

Emodin (purity ≥ 98%) was obtained from Shanghai Sunny Biotech Co., Ltd. (Shanghai, China). Diazinon was obtained from AccuStandard, Inc. (Shanghai, China). The organic solvents utilized in this study, namely, acetonitrile, methanol and hexane, were of HPLC grade (Tedia Company, Fairfield, OH, USA), and all other chemicals used in this study were of analytical grade.

### Experimental fish

Healthy grass carp (males and females, 120 ± 5 g) were obtained from Ganyu Aquaculture Company in Lian Yungang, Jiangsu, China. The fish were maintained in circular fiberglass tanks with flowing seawater. Prior to initiation of this study, eight fish were selected to serve as the negative controls and were analyzed to confirm the absence of emodin and DZN. The fish were allowed to acclimatize for 2 weeks before drug administration and were fed a commercial fish diet free of emodin. The water was recirculated, the degree of aeration was maintained at a constant level, and the temperature was maintained at 25.0 ± 1.0°C. For the experiment, three random groups were cultured. The fish were fasted for 48 h before drug administration. All fish were handled in accordance with the guidelines for the care and use of animals for scientific purposes set forth by the Institutional Animal Care and Use Committee (IACUC) of the East China Sea Fisheries Research Institute at the Chinese Academy of Fishery Sciences in China. The IACUC approved this study within a project of the Special Fund for National Natural Science Foundation of China.

### Oral administration, exposure and sampling

Fish (n = 50) were selected by stratified randomization and then divided into two groups (control group and emodin + DZN group). Emodin (30 mg·kg^-1^ body weight) was forced into the stomach of the fish using a 1-mL syringe with a blunt 12-gauge needle on three consecutive days, and the fish were then cultured in tanks with 0.1 mg·L^-1^ DZN. The control group was orally administered an equal amount of nonmedicated bait and cultured in a tank without DZN. After the last feeding, the fish were anaesthetized using AQUI-S at a concentration of 10 mg·L^-1^ (AQUI-S New Zealand Ltd, Lower Hutt, New Zealand) and processed to obtain nine tissue samples (n = 5) for experimental analysis. Only those individuals who did not regurgitate were used in the experiment. The concentration of DZN in the experiment tanks was consistent maintained at 0.1 mg·L^-1^.

Selected fish (n = 100) were orally administered emodin (30 mg·kg^-1^ b.w.) and exposed to DZN at a concentration of 0.1 mg·L^-1^. Samples were collected at 0.5 h, 1 h, 3 h, 6 h, 12 h, 24 h, 2 d, 3 d, 5 d and 7 d after the administration of emodin. At each time point, five fish were transferred to a sampling tank, anaesthetized with AQUI-S at a concentration of 10 mg·L^-1^ (AQUI-S New Zealand Ltd, Lower Hutt, New Zealand) and processed for tissue collection. Various tissues, including the liver, intestine, muscle, kidney, spleen, brain, fin, skin and gill, were collected, placed in individually marked plastic bags, and frozen at -80°C.

### Sampling for ABC transporter gene profiling RNA extraction

RNA was extracted from the tissues collected at 0.5 h, 1 h, 3 h, 6 h, 12 h, 24 h, 2 d, 3 d, 5 d and 7 d. Specifically, tissue samples stored in RNAlater were isolated using the TRIzol reagent (Invitrogen, USA) according to the manufacturer’s instructions. The RNA was reverse transcribed using a SuperScript Reverse Transcriptase kit (Invitrogen, USA).

#### Primer design and testing

The expression of *ABC* transporter gene transcripts in different tissues was measured by real-time quantitative PCR (qRT-PCR). The *elongation factor 1a* (*EF1α*) gene was used as an internal control. The primers used for the amplification of ABC transporter genes and *EF1α* are listed in Table 1. PCR amplification was performed in a total volume of 15 μl, which contained 1× Maxima^TM^ SYBR Green qPCR Master Mix (Fermentas, USA), 0.2 μM of each primer and 1 μl of normalized template cDNA. The PCR conditions were 95°C for 3 min followed by 40 cycles of 15 s at 95°C, 30 s at 60°C and 20 s at 72°C, and a melting curve was obtained to assess the specificity of the qRT-PCR amplification. Additional cycles were performed by reading the fluorescence value from 60 to 95°C. Sterile deionized water instead of cDNA template was used to generate the negative controls. Each experiment was repeated in triplicate. A standard curve of each pair of primers was generated to estimate the amplification efficiencies based on known quantities of cDNA (4-fold serial dilutions corresponding to cDNA transcribed from 100 to 0.1 ng of total RNA). All calibration curves exhibited correlation coefficients higher than 0.99, and the corresponding real-time PCR efficiencies (E) were higher than 0.95. The expression of ABC transporter genes was analyzed using the 2-ΔΔCT method [24].

**Table 1:** qRT-PCR primers used for the assessment of gene expression in grass carp.

Gene	Primer sequence (5′-3′)	GenBank accession number	Ta (°C)	Ref.
EF1α-F1	CGCCAGTGTTGCCTTCGT	GQ266394	60.0	[39]
EF1α -R1	CGCTCAATCTTCCATCCCTT			
ABCB1-F	CGTTCCTCAAGGTGATGGCT	DQ059072	60.0	[5]
ABCB1-R	GGCTGCATTGCACCATTGAT			
ABCB11-F	CTGGTCAGACACTGGCCTTT	GQ911570	60.0	[5]
ABCB11-R	CAGGAAAGACACGTTGACGC			
ABCC1-F	ATCCGTGAGAGTGACCAG	GQ911567.1	60.0	[5]
ABCC1-R	CAAATGACACAATGAAGTTTCC			
ABCC2-F	CCTGGTTGGCTTGTCTATATCC	KC256782.1	60.0	[40]
ABCC2-R	CTCGCTGTATTCACTCACTCTC			
ABCG2-F	TCATGAAGCCGGGTCTCAAC	XM_019117679.1	60.0	[39]
ABCG2-R	AGACCTGCAGGGTCCTTTCT			

### Determination of the accumulation of emodin and DZN in tissues of grass carp

#### Sample preparation

Calibration standards of emodin (5 ng, 50 ng, 500 ng, 2500 ng, 4000 ng and 5000 ng) and DZN (0.625 ng, 6.25 ng, 62.5 ng, 312.5 ng, 500 ng, 625 ng) were prepared. Data acquisition and integration of the chromatograms were performed using Xcaliber 3.0 (Thermo). The chromatographic data were analyzed by linear least-squares regression with a weighting of 1/X^2^, which generated a six-point calibration curve of the area ratios for emodin and DZN, respectively. The calibration curves for emodin and DZN were linear over the range of 5.00 to 5,000 ng·mL^-1^ (R^2^ > 0.98) and 0.625 to 625 ng·mL^-1^ (R^2^ > 0.99), respectively.

Samples of grass carp tissues, including the liver, intestine, muscle, kidney, spleen, heart, brain, fin, skin and gill, were collected. The tissue samples (1.0 g) were thawed and triturated using a mortar and pestle. Hexane/acetonitrile (1.0 mL) was added to the samples, and the mixtures were vortexed for 2 min and then centrifuged at 13,000 × *g* for 5 min. The supernatant was transferred to a clean centrifuge tube, and the removed aqueous layer was re-extracted twice using 0.2 mL of acetonitrile. The combined acetonitrile solution was evaporated to dryness using a vortex evaporator. The mixture was shaken vigorously and transferred to a 5-mL centrifuge tube. After centrifugation at 4,150 × *g* for 5 min, the bottom layer of liquid was filtered through a 0.22-μm disposable syringe filter and then analyzed by high-performance liquid chromatography (HPLC) and liquid chromatograph-mass spectrometry (LC-MS).

#### Instrumentation and conditions

An UltiMate 3000 RS system (Thermo Fisher Scientific, USA) consisting of a double pump, an autoinjector, a column temperature tank, and an ultraviolet light detector was used for the HPLC analysis. The operating conditions were as follows: Hypersil GOLD C18 columns (100 mm × 2.1 mm i.d., 1.9-μm particle size); flow rate, 0.5 ml·min^−1^; column temperature, 20°C; and mobile phase, 0.01 mM aqueous ammonium formate: acetonitrile solution (30:70, v/v). The LC effluent was split and introduced into the inlets of two mass spectrometers in parallel. The two mass detectors were equipped with an ESI and operated in the positive and negative ionization modes (each detector was operated in one of the modes).

The LC-MS separations were performed using a Hypersil GOLD (100 mm × 2.1 mm i.d., 1.9-μm particle size) analytical column with a C18 SecurityGuard cartridge. The eluent flow rate was set to 0.5 mL·min^−1^, and the column was maintained at 20°C. A TSQ Quantum triple quadrupole mass spectrometer (Thermo Fisher Scientific, USA) equipped with an electrospray ionization interface operating in both the positive ion (PI) and negative ion (NI) modes according to the preferential ionization of each analyte was used. The drying and nebulizing gas for the LC-MS analysis was produced in situ by a nitrogen generator fed compressed air at 7 bar. The optimized ESI parameters were a drying gas flow rate of 12 L·min^−1^, a drying gas temperature of 300°C, a nebulizer gas pressure of 40 psi, and a spray voltage of 4002 V-4001 V (PI/NI).

### Data analyses

All statistical analyses were performed using SPSS 16.0 (SPSS, Chicago, IL, USA), and the experimental data are presented as the means ± standard deviations (SDs) (n ≥ 3). All qRT-PCR data are presented as the means ± SEs (standard errors). Comparisons among different tissues were analyzed using one-way analysis of variance (ANOVA). The statistical significance of the differences in the ABC transporter genes after emodin treatment combined with DZN exposure was estimated by two-way ANOVA followed by Duncan’s multiple range tests. Values of *p* < 0.05 were considered statistically significant in all experiments.

## Results

### Gene transcript abundance of ABC transporters in normal individuals

**Fig 1:**
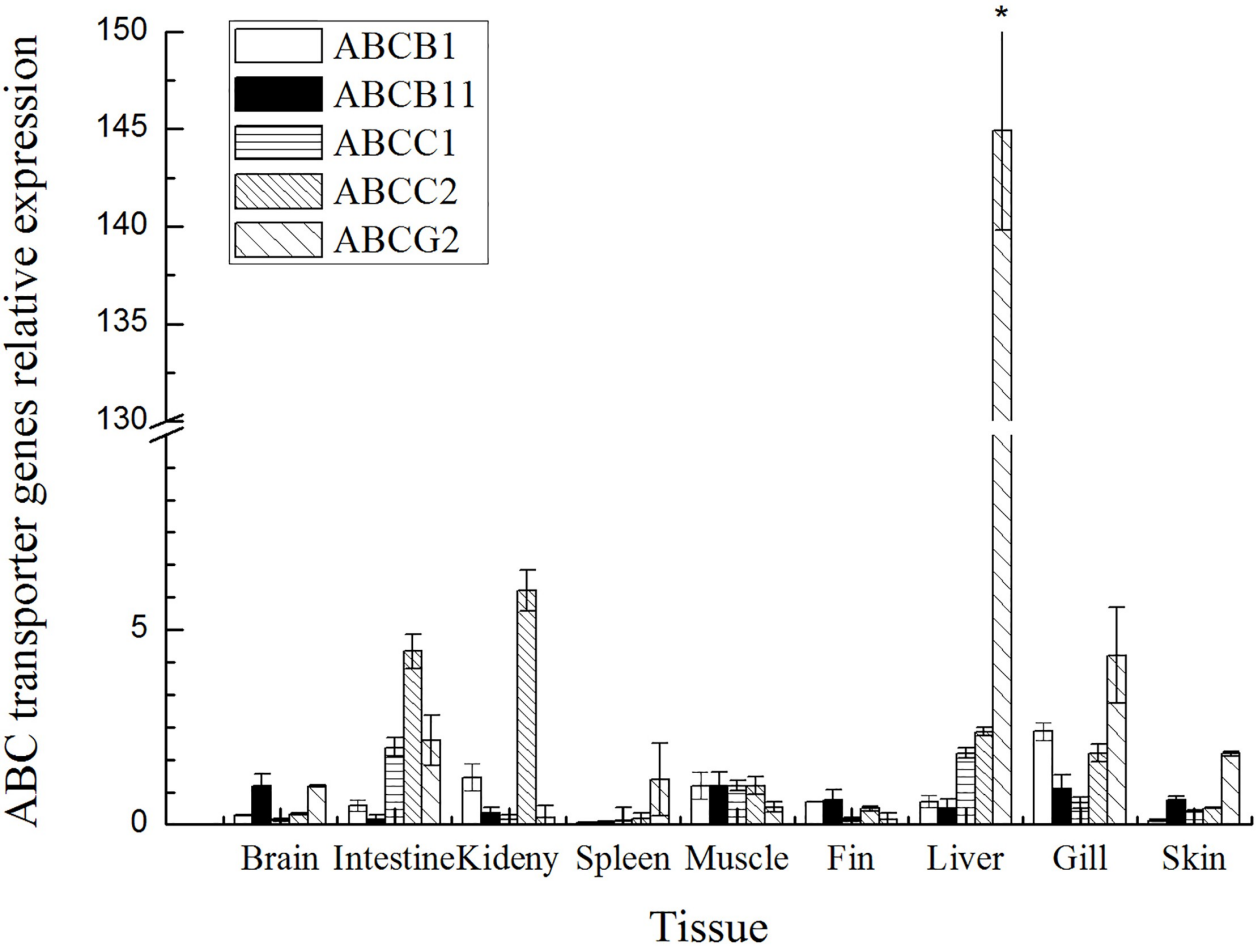
ABC transporter genes expressed in normal tissues (n = 5).

The mRNA expression levels of the *ABCB*1, *ABCB*11, *ABCC*1, *ABCC*2 and *ABCG*2 genes in ten tissues of healthy grass carp, including the liver, intestine, muscle, kidney, spleen, heart, brain, fin, skin and gill tissues, was analyzed, and a summary of the ABC transporter gene expression results is provided in Fig 1. The highest level of the *ABCB*1 gene was found in the gill, kidney, muscle and liver; moderate expression was detected in the fin, intestine and heart; and the lowest expression of this gene was observed in the skin and spleen. The highest *ABCB*11 expression level was found in the muscle, gill, brain and skin; moderate expression was detected in the fin, liver, kidney and intestine; and the lowest expression was observed in the heart and spleen. Most notably, *ABCC*1 was expressed at high levels in the intestine, muscle, liver and gill, at lower levels in the skin brain, and fin, and at the lowest levels in the heart, spleen and kidney. The highest *ABCC*2 expression level was found in the kidney, intestine, liver and gill, whereas lower expression of this gene was detected in the muscle, fin, skin and brain, and the lowest expression level was observed in the spleen and heart. The *ABCG*2 gene was expressed at its highest levels in the liver (*p* < 0.05), gill, heart and intestine, at lower levels in the skin, spleen, brain and muscle, and at the lowest levels in the kidney and fin. ABC transporter genes show differential expression in the various tissues of grass carp.

### Gene transcript levels in various tissues after emodin treatment combined with DZN exposure

**Fig 2:**
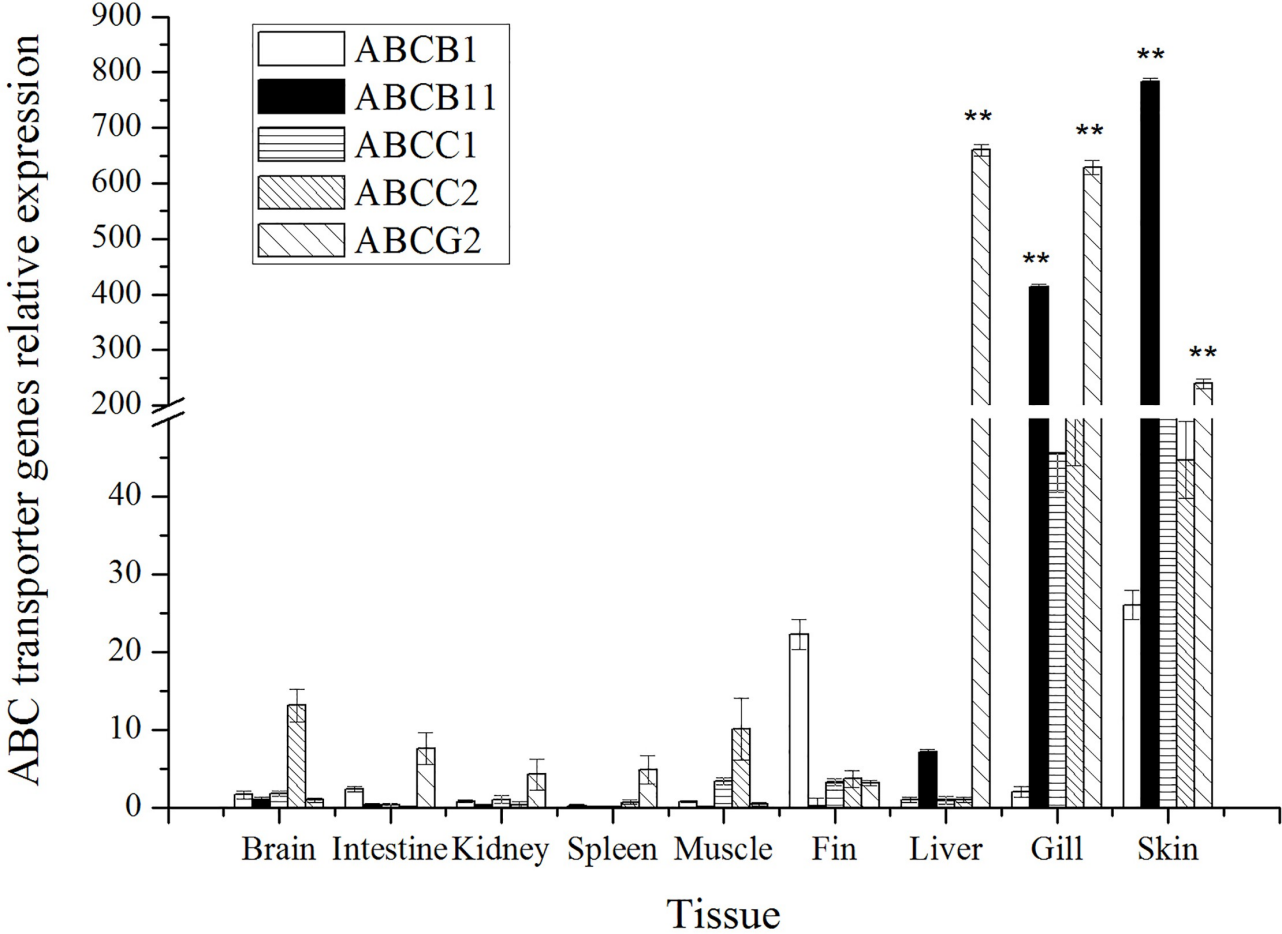
Expression of ABC transporter genes in tissues of grass carp treated with emodin combined with DZN exposure (n = 5).

The mRNA expression profiles of the ABC transporter genes in various tissues of grass carp after three consecutive days of treatment with emodin combined with DZN exposure were investigated. The expression of ABC transporter genes was different in the different tissues; in particular, the skin, liver and gill tissues showed significantly higher expression levels than the other tissues (Fig 2). The expression of the *ABCB*1 and *ABCB*11 genes was higher in the skin (26-fold and 109-fold (*p* < 0.01), gill (2-fold and 57-fold (*p* < 0.01), respectively) and liver (1-fold and 7-fold, respectively) than in the other tissues. The *ABCC*1 gene was more highly expressed in the skin (127-fold) than in the liver (*p* < 0.01). The *ABCC*2 gene was more highly expressed in the gill (50-fold) and skin (45-fold) than in the liver. Higher levels of *ABCG*2 expression were found in the skin (659-fold, *p* < 0.01), gill (628-fold, *p* < 0.01) and liver (659-fold, *p* < 0.01) compared with brain tissue. The ABC transporter family of genes showed significant differential expression in various tissues after treatment with emodin combined with DZN exposure. In particular, the skin, gill and liver tissues were significantly affected by emodin combined with DZN exposure.

### Gene transcript levels in grass carp at various time points after emodin treatment combined with DZN exposure

**Fig 3:**
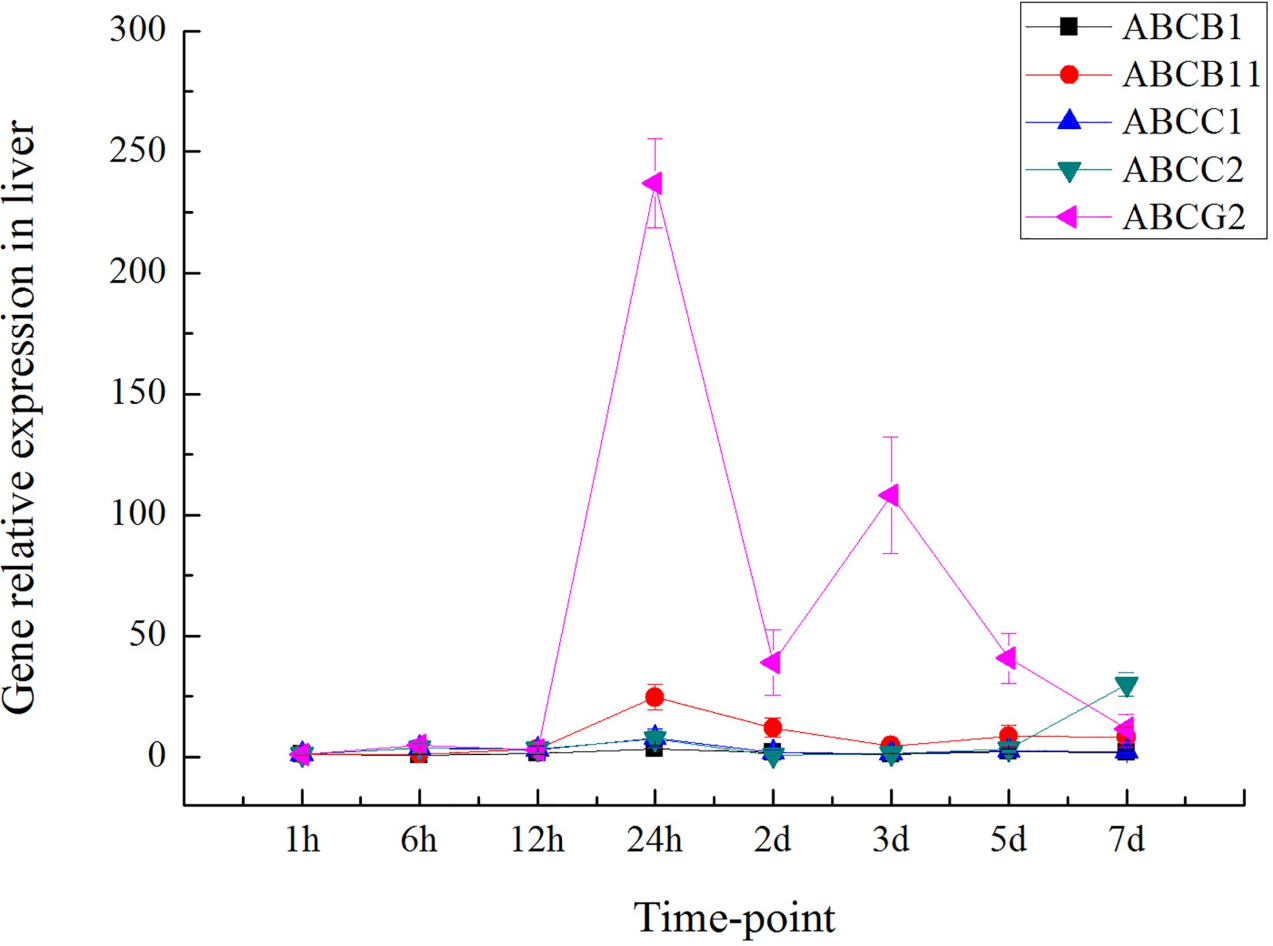
Expression of ABC transporter genes in the liver of grass carp at different time points after emodin treatment combined with DZN exposure (n = 5).

**Fig 4:**
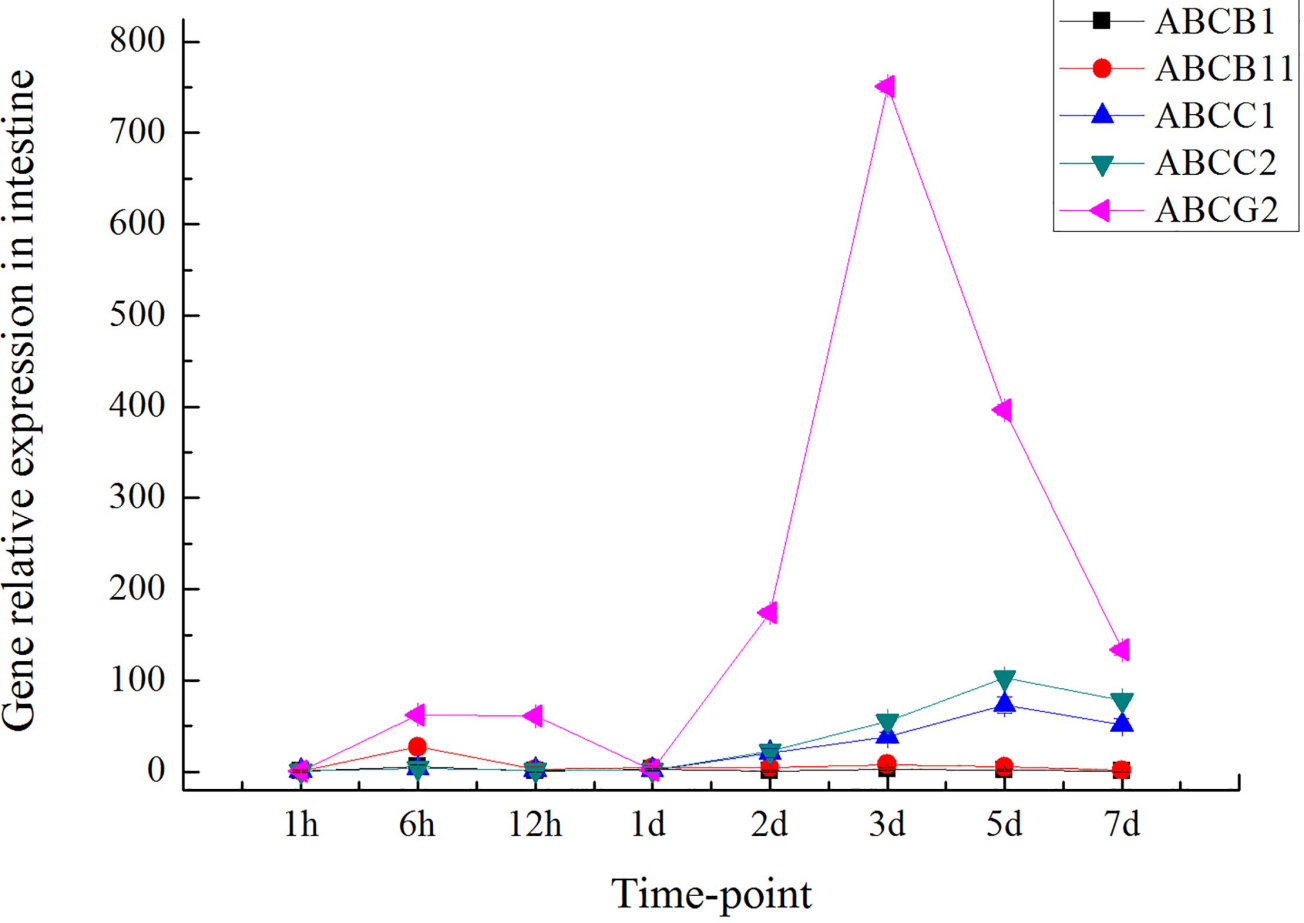
Expression of ABC transporter genes in the intestine of grass carp at different time points after emodin treatment combined with DZN exposure (n = 5).

The expression levels of ABC transporter genes in the liver and intestinal tissues of grass carp after 0.5 h, 1 h, 3 h, 6 h, 12 h, 24 h, 2 d, 3 d, 5 d and 7 d of treatment with emodin combined with DZN exposure were measured (Figs 3 and 4). As shown in Fig 3, the transcript levels of both *ABCB*1 and *ABCB*11 showed similar trends in the liver in the treatment emodin combined with the DZN exposure group. The transcript levels of both *ABCC*1 and *ABCC*2 also displayed similar trends at different time points in liver tissue. The highest levels of *ABCB*1, *ABCB*11, *ABCC*1, *ABCC*2 and *ABCG*2 were found in liver tissue 24 h after emodin treatment combined with DZN exposure. Furthermore, the expression of *ABCG* in liver tissue was higher than that of *ABCB*1, *ABCB*11, *ABCC*1 and *ABCC*2. In intestinal tissue, however, different expression patterns were found for the ABC transporter genes at various time points (Fig 4). The highest levels of *ABCC*1 and *ABCC*2 were found after 24 h of treatment, whereas the peak *ABCB*1 and *ABCB*11 expression levels were detected after 6 h of treatment. The highest *ABCG*2 expression level was observed after 3 d of treatment with emodin combined with DZN exposure. Furthermore, *ABCG2* was expressed at significantly higher levels than the other ABC transporter genes.

### Tissue accumulation of emodin and DZN in various tissues of grass carp

**Fig 5:**
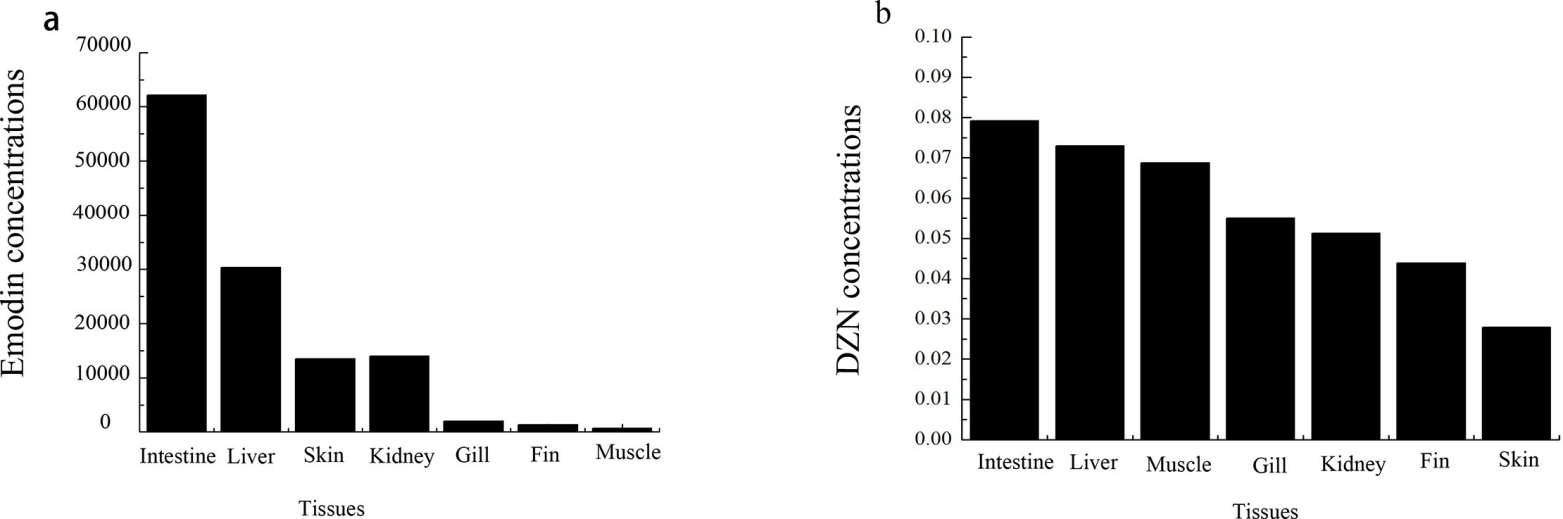
Emodin (a) and DZN (b) concentrations in various tissues of grass carp (n = 5).

The concentrations of emodin and DZN in the tested tissues were calculated using the corresponding calibration curves. As shown in Fig 5, the highest emodin concentrations were found in the intestine (62.19 μg·ml^-1^) and liver (30.37 μg·ml^-1^), whereas moderate concentrations were detected in the kidney (14.01 μg·ml^-1^) and skin (13.49 μg·ml^-1^), and the lowest concentrations were found in the gill (1.99 μg·ml^-1^), fin (1.35 μg·ml^-1^) and muscle (0.68 μg·ml^-1^). Higher DZN concentration was found in the intestine (0.079 ng·ml^-1^) and liver (0.073 ng·ml^-1^) than in the other tissues.

### Accumulation of emodin and DZN in the liver and intestine of grass carp over time

**Fig 6:**
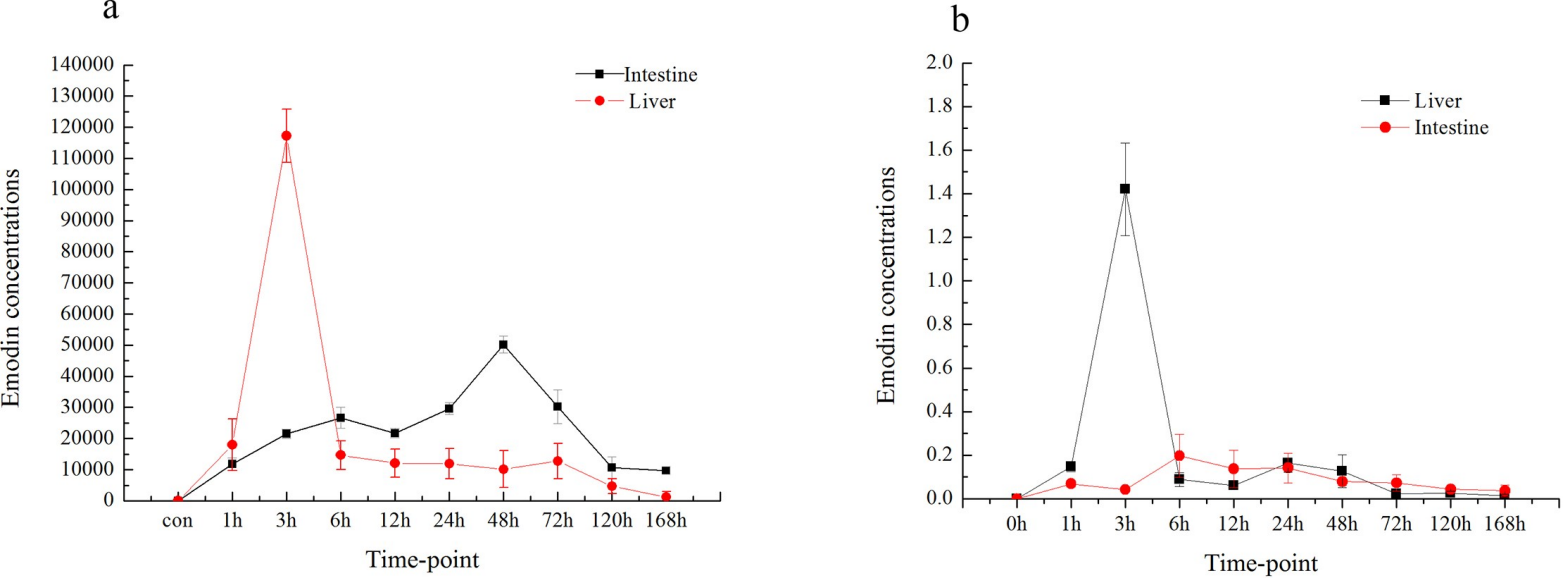
Emodin (a) and DZN (b) concentrations in the liver and intestine of grass carp at different time points (n = 5).

The emodin and DZN concentrations in the liver and intestinal tissues of grass carp at different time points after the initiation of treatment were investigated (Fig 6). The peak emodin concentration in the liver (50.18 μg·ml^-1^) was detected 48 h after the oral administration of emodin. However, the peak emodin concentration in intestinal tissue (117.24 μg·ml^-1^) was found 1 h after the oral administration of emodin. The peak DZN concentrations in the liver and intestinal tissue (1.42 ng·ml^-1^ 0.20 ng·ml^-1^) were found after 3 and 6 h of DZN exposure, respectively.

## Discussion

Aquatic animals are constantly exposed to natural or anthropogenic toxicants in their environment, and in these animals, the ABCB, ABCC and ABCG subfamilies play important roles in the excretion of xeno- and endobiotics and their metabolites. The tissue distribution of the ABC transporters shows differences in aquatic animals [25]. A previous study determined the expression of ABC transporter genes and showed that various xenobiotics are found in several tissues of rainbow trout [26], and the expression of ABC transporters has also been detected in Nile tilapia after exposure to benzo(a)pyrene [5]. However, few studies have focused on the expression of ABC transporter genes that are influenced by xenobiotics in aquatic organisms. Importantly, there is limited information on how Chinese herbal medicines regulate the expression of ABC transporters and influence the ability of an aquatic animal to transport environmental toxicants. In this study, we found that the expression of ABC transporter genes in grass carp was altered by emodin treatment combined with DZN exposure.

### Distribution of ABC transporter genes

ABCB1 is an apical membrane transporter located in the kidney, liver, intestinal, gill and capillary endothelia, where it contributes to the formation of the blood-brain barrier and functions to protect against xenobiotics and cellular toxicants [1]. Previous studies have determined the expression of ABCB1 in a wide range of tissues. The results of this study suggest a similar role for ABCB1 in mediating the efflux of xenobiotics from the gill, liver and intestinal tissues of fish. ABCC, which belongs to the ABCC subfamily, is localized to the basolateral cellular surface, which in certain tissues results in the efflux of its substrate into the blood [3]. ABCC1 and ABCC2 are well-studied drug efflux transporters in aquatic animals. ABCC2 plays an important role in the hepatobiliary elimination of drugs and toxins as well as their metabolites. This study found that ABCC1 and ABCC2 are expressed at high levels in the intestinal, muscle, liver and gill. ABCG2, which belongs to the ABCG subfamily, plays a major role in the efflux of xenobiotics in mammals [27]. A previous study found that ABCG2 is highly expressed in gonads and moderately expressed in the distal part of the intestinal, in the kidney and in brain tissues of rainbow trout [26]. However, in this study, the highest ABCG2 expression levels were found in the liver and gill, and the lowest expression levels of this gene were detected in the kidney and fin. This result does not agree with that found for zebrafish, which exhibits no ABCG2 expression in the kidney [28]. These findings indicate that the expression of ABCG2 in particular tissues could be species-specific in fish.

In this study, the expression of ABC transporter genes was measured and analyzed, and the results indicated the distributions of these genes in various tissues of grass carp after emodin treatment combined with DZN exposure. The genes encoding ABC transporter proteins were expressed at relatively high levels in the liver, intestinal, gill and skin. These genes have been increasingly recognized for their ability to modulate the absorption, distribution, metabolism and elimination of xenobiotics in tissues.

### Characteristics of the ABC transporter distribution

The liver plays major roles in the detoxification of endogenous and foreign compounds, including many organic chemicals that could be considered environmental pollutants to aquatic animals [29]. The elimination of drugs and toxins or their metabolites might involve efflux transport by ABC transporters, which would result in biliary excretion from the liver. The importance of the biliary route of excretion is illustrated by the wide range of compounds found in fish bile, which includes agricultural compounds such as pesticides [30]. Data on the mRNA expression of ABC transporters in the liver are available for a number of fish species. For example, the *ABCB*1 gene of turbot (*Scophthalmus maximus*) has been cloned, and its RNA has been detected in the brain, intestinal, kidney and liver [31]. In rainbow trout, ABCC1, ABCC4 and ABCC5 expression is low in the liver (< 5 copies/ng of total RNA) [26]. In this study, the highest expression of ABCG was found in the liver, which indicated that ABCG was more significantly influenced by emodin combined with DZN than the other ABC transporter genes. The ABC transporter genes showed differential expression in the liver of grass carp, which implies that different ABC transporter genes might play different roles in fish species.

The intestine has multiple functions that primarily include digestion and nutrient absorption, maintenance of the acid-base balance and immune function in animals [32, 33]. This tissue is also major site for the expression of ABC transporters, which limit the absorption of drugs, such as transport substrates [34]. However, the available information on the expression and activity of ABC transporters in the intestines of fish is limited. For example, ABCB1, ABCC2, ABCC3 and ABCG2 are highly expressed in the intestine of rainbow trout, whereas ABCC1, ABCC4 and ABCC5 are present in this tissue at low transcription levels [26]. In this study, ABCC1, ABCC2 and ABCG2 were found at high expression levels in the intestines of grass carp, whereas ABCB1 and ABCB11 were observed at low transcription levels. After emodin treatment combined with DZN exposure, the expression of ABCC1, ABCC2 and ABCG2 mRNA was higher than that of the other ABC transporter genes, which indicated that these genes were more highly upregulated by emodin than the other ABC transporter genes.

The gills are potentially characterized by a high influx of xenobiotics, which could be explained by their large surface area and constant contact with the external environment. The gills constitute a main site of gas exchange, osmoregulation and the excretion of nitrogenous waste products in aquaculture animals [35]. The gills also represent a dominant site for both the absorption and the elimination of xenobiotics [36]. A previous study on rainbow trout revealed high ABCC2 and ABCC3 mRNA expression in the gills when the data were reported as copy numbers per mass unit of total RNA, whereas ABCC3 and ABCG2, and to a lesser extent ABCC5, presented the highest transcription level based on quantification relative to the reference gene *EF1α* [26]. In this study, the mRNA expression of ABCB1, ABCB11, ABCC1, ABCC2 and ABCG2 in the gills of grass carp was measured. The transcription levels of ABC transporter genes in the fish treated with emodin and exposed to DZN were higher than those in normal individuals. This finding suggests that ABC transporter genes might be critical for xenobiotic excretion in the gills. The ABC transporters were found to be expressed at different levels in different tissues, and their major functions based on the differential tissue expression of the tested genes are discussed above. In particular, the liver, intestinal and gills are the major tissues for metabolic detoxification.

### Specification of the ABC transporter

ABC transporter proteins transport a wide spectrum of substances and exhibit major differences in their transport mechanisms and types of substrates. The specificity of the substrates and tissue distributions of different ABC transporters are mainly caused by differences in the structure of their NBD. For example, ABCB1 and ABCB2 belong to the ABCB subfamily and share 40% amino acid identity with each other. In contrast, ABCB1 and ABCG2 belong to separate subfamilies of the ABC family and share very limited amino acid identity (< 20%) with each other [37]. The tissue distributions of ABCB1 and ABCB2 differ from each other, but collectively, their isoforms have the same distributions in fish as in mammals, which suggests that these proteins share a similar physiological function across species [3]. ABCB1, ABCB11, ABCC1, ABCC2 and ABCG2 are known to have wide spectra of substrates, which can be activated by environmental pollutants. However, drugs and toxins are transformed by different ABC transporter subfamilies.

In various tissues of grass carp, the ABCB11 and ABCG2 transcription levels were found to be significantly upregulated compared with those of other transporter genes after emodin treatment combined with DZN exposure. We thus hypothesize that ABCG2 and ABCB11 might play important roles in the metabolism and transport of emodin and DZN in grass carp. This study provides valuable insights into ABC transporters that are influenced by emodin and DZN in grass carp. However, the functions and relationships among mRNA expression, transporter protein abundance and enzyme activity of ABC transporters might not be direct and linear, and further study is thus needed.

### Relationship between ABC transporters and accumulation of emodin and DZN over time

The ABC transcript levels in grass carp treated with emodin combined with DZN exposure at various time points were measured. The peak ABC transporter transcript levels in the liver and intestine were found 24 h and 3 d after emodin treatment combined with DZN, respectively. Furthermore, the transcription level of ABCG2 was higher than that of the other ABC transporter genes, which showed that ABCG2 might play an important role in transport processes in grass carp treated with emodin combined with DZN exposure. We determined that the liver plays a major important role in drug metabolism and that the intestine is involved in drug transport and absorption. Therefore, the peak ABC transcript levels were observed in the liver and intestine. Furthermore, ABCG showed higher expression than the other ABC transporter genes tested in this experiment, which indicated that ABCG is the major ABC transport gene for the transport of emodin and DZN in grass carp. This phenomenon might be explained by the prominent expression of ABCG2 in the apical membranes of polarized epithelial cells in different organs and tissues involved in absorption (small intestine), distribution (blood brain barrier) and elimination (liver), which indicates an important role for ABCG2 in the excretion of xenobiotics [38].

In conclusion, this study provides insights into the distribution patterns of a series of potentially toxicology-related ABC transporters in fish. Importantly, this study also indicates that the transcription level of ABC transporters, particularly *ABCG2*, plays an important role in regulating the metabolism process of emodin in grass crap. Furthermore, the accumulation of emodin in the liver and intestine of grass carp indicates that emodin has greater affinity for the liver and intestine than for the other tissue of grass carp. Based on these findings, emodin might play a significant role in the regulation of ABC transporters and thereby in the transport of environmental pollutants in aquatic animals.
